# Students’ perspectives on research and assessment of a model template designed to guide beginners in research in a medical school in Cameroon

**DOI:** 10.1186/s12909-014-0269-y

**Published:** 2014-12-21

**Authors:** Joshua Tambe, Jacqueline Ze Minkande, Boniface Moifo, Robinson Mbu, Pierre Ongolo-Zogo, Joseph Gonsu

**Affiliations:** Faculty of Medicine and Biomedical Sciences, The University of Yaoundé 1, PO Box 1839, Yaoundé, Cameroon; Center for the Development of Best Practices in Health, Yaoundé, Cameroon; Doctoral Research Unit, The University of Yaoundé 1, Yaoundé, Cameroon

**Keywords:** Medical trainees, Novel self-help template

## Abstract

**Background:**

Research activities for medical students and residents (trainees) are expected to serve as a foundation for the acquisition of basic research skills. Some medical schools therefore recommend research work as partial requirement for certification. However medical trainees have many difficulties concerning research, for which reason potential remedial strategies need to be constantly developed and tested. The views of medical trainees are assessed followed by their use and appraisal of a novel “self-help” tool designed for the purposes of this study with potential for improvement and a wider application.

**Methods:**

This study was a cross-sectional survey of volunteering final-year medical students and residents of a medical school in Cameroon.

**Results:**

This study surveyed the opinions of a total of 120 volunteers of which 82 (68%) were medical students. Three out of 82 (4%) medical students reported they had participated in research activities with a publication *versus* 10 out of 38 residents (26%). The reported difficulties in research for these trainees included referencing of material (84%), writing a research proposal (79%), searching for literature (73%) and knowledge of applicable statistical tests (72%) amongst others. All participants declared the “self-help” tool was simple to use, guided them to think and better understand their research focus.

**Conclusion:**

Medical trainees require much assistance on research and some “self-help” tools such as the template used in this study might be a useful adjunct to didactic lectures.

## Background

Research work is mandatory before graduation from medical schools in Cameroon unlike other developing countries such as Brazil [1]. Medical students are expected to carry out research and defend their work publicly in the final year of studies. A “Doctor of Medicine” thesis is partial requirement for undergraduate certification while a dissertation is recommended for specialist qualification. Such exercise is expected to serve as a foundation for the acquisition of basic research skills. Medical students can carry out basic research and publish in peer-reviewed journals. Although an increase in articles by medical students has been reported over the years, these studies are rarely cited [2]. There is nevertheless more encouragement for medical students to be engaged in research [3,4].

The Faculty of Medicine and Biomedical Sciences of The University of Yaoundé 1 is the oldest medical school in Cameroon and in Central Africa. Students are expected to have a mastery of both English and French. Despite efforts to endow students with basic research skills through the conduct of research and a public defense over the years, many problems persist. Medical trainees often do not really know where to begin once a research topic has been handed to them (as is often the case) by a prospective research director. Most have never written an academic or scientific paper and do not know how to go about one. The tendency for some is to attempt to reproduce previous documents, copying entire sections ‘as is’ without any consideration of relevance.

Continuous teaching of research methods has been reported to have a positive impact on medical students [5,6]. Different methods and models to facilitate comprehension need to be continually tested and employed as there is no “magic” formula for every student. To help medical trainees think independently and systematically is the reason why a novel tool was developed and used in this study. Needs assessment will also help to identify aspects that may require more attention. In this study the views of medical trainees are assessed followed by their use and appraisal of a novel “self-help” tool designed for the purposes of this study with potential for improvement and a wider application.

## Methods

This study was a cross-sectional survey of volunteering final-year medical students and residents of the Faculty of Medicine and Biomedical Sciences of The University of Yaoundé 1 engaged in an end-of-course research work. Eligible participants were contacted by one of the authors (JT) in the university campus or hospitals affiliated to the university. After explanation of the study to potential participants consent to participate was sought verbally.

**Figure 1:**
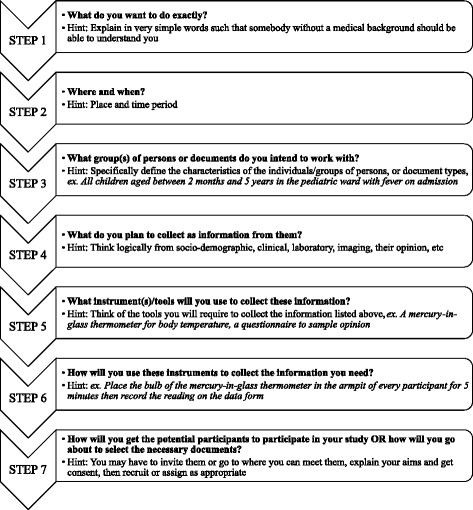
**Presentation of model template.**

The questionnaire had two sections. The first section contained questions on if the respondents had previously participated in research activities, written any scientific paper or academic research project, the difficulties they had or were having and how they sought it out or were planning to do so. Their views on the necessity of research before leaving school were also ascertained. The second section contained a novel “self-help” tool designed by the principal investigator with intention to help guide beginners in research “think through” their research projects. Hereafter referred to as a model template, this tool consists of a series of seven steps with questions and hints arranged in a particular sequence. Blank sheets of paper were attached to the questionnaire for use with the template. The present format of the template is the result of pre-testing using volunteers from the previous batch of final-year medical trainees from the same institution. This template is presented in Figure 1.

Data collection lasted from October 2013 to February 2014, time period during which the study target population was not expected to be on vacation according to their academic year timetable. Some participants responded and handed back the questionnaire and attached blank sheets immediately to the principal investigator, whilst others handed them later. The participants tested the model template each with their research topics and wrote down their ideas on the blank sheets of paper provided (these they kept for their personal use). Assistance was readily offered by the principal investigator when requested. Participants then responded to some more questions on the effect the use of the template had on their understanding of their research work. Authorization for the study was granted by the Ethics Committee of the Faculty of Medicine and Biomedical Sciences, Yaounde. Data are presented as counts and percentages with the use of tables where necessary.

## Results

A total of 120 medical students and residents across the different specialties offered by the university participated in the survey. Of these, 82 (68%) were medical students. Close to 150 medical students and about 50 to 60 residents are admitted each year into this medical school. However close to a quarter of the number of final-year trainees travel to another town or country for internship. Responses were available from 120 participants from an estimated 158 available (response rate = 76%). Table 1 summarizes some aspects of the first part of the survey.

**Table 1:** **Summary of some items of the first part of the survey**

	**Medical students, n = 82**	**Residents, n = 38**
*Have you ever participated in research activities?*	Yes = 4 (5%)	Yes = 35 (92%)
	No = 78 (95%)	No = 3 (8%)
*If yes, what was your role?*	Volunteer	Investigator
*Have you ever published an article in a scientific journal?*	Yes = 3 (4%)	Yes = 10 (26%)
	No = 79 (96%)	No = 28 (74%)
*Have ever written a thesis or dissertation?*	Yes = 0	Yes = 33 (87%)
	No = 82 (100%)	No = 5 (13%)

Thirty-three residents (87%) declared they had written a thesis at undergraduate level. The difficulties in research that both medical students and residents admitted they had or were having included the following (n = 120):

- Referencing material using a consistent style (n = 88, 73%)
- Did not know how to write a research proposal (n = 83, 69%)
- Did not know where to get material for literature review (n = 77, 64%)
- Did not know which statistical tests were applicable to their research (n = 76, 63%)
- Could not make a questionnaire or draft a form for data recording (n = 68, 57%)
- Did not know how to write up the methodology section (n = 61, 51%)

All participants stated having received lectures on research methods before they started out on their research projects. Sixty-seven medical students (82%) remarked that the lectures they received were not helping them much as they still had to look for what they specifically needed elsewhere (the internet, books on statistics and epidemiology, theses and dissertations in libraries). However, 35 out of 38 (92%) residents did find the lectures they received on research methods while in residency useful.

Table 2 summarizes the perspectives of the respondents on the necessity of research before leaving medical school and shows that medical students were more positive.

**Table 2:** **Response of participants on the necessity of research**

**Medical students, n = 82**	**Residents, n = 38**
Yes = 56 (68%)	Yes = 13 (34%)
Not quite = 24 (29%)	Not quite = 19 (50%)
No = 2 (3%)	No = 6 (16%)

The respondents’ assessment of the model template is presented in Table 3. Many reported it provided guidance, was quite easy to use and they might readily recommend its use.

**Table 3:** **Assessment of model template**

	**Medical students, n = 82**	**Residents, n = 38**
*Does this template guide you as to how you can proceed to collect data for your study?*	Yes = 82 (100%)	Yes = 38 (100%)
	Partially = 0	Partially = 0
	No = 0	No = 0
*Does the template permit you to better understand and explain your study?*	Yes = 82 (100%)	Yes = 37 (97%)
	Partially = 0	Partially = 1 (3%)
	No = 0	No = 0
*How did you find its use?*	Very easy = 2(2%)	Very easy = 11(29%)
	Quite easy = 68(83%)	Quite easy = 21(55%)
	Moderate = 12(15%)	Moderate = 6(16%)
	Difficult = 0	Difficult = 0
	Very difficult = 0	Very difficult = 0
*Would you recommend it to someone else?*	Yes = 82 (100%)	Yes = 38 (100%)
	Not sure = 0	Not sure = 0
	No = 0	No = 0

## Discussion

Lectures on research methods are incorporated in the curricula at undergraduate and post-graduate levels in this medical school. Lecturers change with time with an expected change in the content and style of the lectures. These lectures are sometimes organized as seminars. In this university medical trainees mandatorily carry out research and defend a thesis or dissertation as partial fulfillment for certification counting up to 50% of the final year score. Despite this compulsory research exercise many of these research projects are not published. About half of the respondents reported they did not think the research exercise was necessary. Frishman reported a similar finding [7] and Siemens et al. concluded from their study that medical students with a previous degree were the ones likely to be more motivated concerning research [8]. Given that the residents had worked for at least two years before enrolling for specialization as was required, it can be inferred that probably the knowledge and skills initially acquired during undergraduate research were not used. This lack of interest in research by medical trainees is not new [9] and some reported contributory factors include the lack of internet facilities and the consideration that research was useless [10].

The stated difficulties in initiation of research were similar for both groups of trainees. This suggests a global insufficiency in the research training methods or possibly a complete lack of interest or motivation on the part of the trainees despite the well-meaning efforts of academic staff. Residents however appreciated lectures received on research methods. This could be due to the fact that they had been involved with research at undergraduate level.

Obviously many students are not actively supervised in their research and so have to fend for themselves. This may be due to several factors such as insufficient time dedicated by over-burdened clinicians to supervise students’ research, increasing workload for academics, low levels of faculty staffing and the general lack of a strong research background in some medical schools. Seemingly the existing training methods do not provide trainees with sufficient competence. Bearing in mind that a good number of final-year medical trainees are novices in research, they will eventually require maximum possible supervision. While hoping for a definite solution to these problems, a self-help tool can assist beginners to get started in their research. The template presented in this paper has been used for this purpose by the authors and is promising. It is often exciting to be involved in research as a final-year medical student because this is a completely new experience from the practice of clinical medicine [11]. However there are many hurdles to overcome and these are sufficient to deter many.

The place of research as a source of scientific knowledge is undisputable. Whether the knowledge and skills will be further harnessed or not, there is the need to continue to educate those interested in the medical sciences on scientific methods, how research generates knowledge and can guide medical practice. This template was developed and tailored to assist medical trainees to think constructively whatever their research focus and write down their own thoughts. The participants in this study used this model template and approved of its guidance and clarity.

Some factors however could have influenced the opinions and views reported in this work. Just one batch of final-year trainees was surveyed. The findings may therefore be specific to this cohort of trainees. Also different lecturers give lectures on research methods in this medical school as the years unfold, leaving room for changes in approach. Furthermore teaching methods are subject to review, and continuing medical education strategies such as seminars and symposia on research are sometimes held in this institution. It is possible for an entire batch to have missed out on these. It is worth noting that no comparison group was employed in the testing of the proposed template, the study volunteers were not blinded to the outcome and reporting bias is likely to have occurred.

The template is intended to be a guide to assist the amateur researcher think, assemble ideas and write down in their own words what they intend to do. It is not a sequence to be memorized, and its use should greatly facilitate the writing of a research proposal. We believe that if the beginner in research can elaborate a strategy that permits them to correctly collect all the data needed for their study, then the essential would have been done. Assistance in data analysis and some other technical aspects can always be obtained from experts. In our opinion the use of such a tool coupled with didactic lectures on research methods might further simplify the learning process and make the initial research experience enjoyable.

## Conclusion

This model template was found to be useful by beginners in research (for whom it was intended) and so therefore can be further tested, improved and put at their disposal. We hope it serves as an adjunct to facilitate comprehension and we discourage any attempt to use it to replace didactic lectures on research methods.
